# The multi-country PROMOTE HIV antiretroviral treatment observational cohort in Sub-Saharan Africa: Objectives, design, and baseline findings

**DOI:** 10.1371/journal.pone.0208805

**Published:** 2018-12-13

**Authors:** Taha E. Taha, Nonhlanhla Yende-Zuma, Jim Aizire, Tsungai Chipato, Lillian Wambuzi Ogwang, Bonus Makanani, Lameck Chinula, Mandisa M. Nyati, Sherika Hanley, Sean S. Brummel, Mary Glenn Fowler

**Affiliations:** 1 Johns Hopkins Bloomberg School of Public Health, Baltimore, MD, United States of America; 2 Centre for the AIDS Programme of Research in South Africa (CAPRISA), Nelson R Mandela School of Medicine, University of KwaZulu-Natal, Durban, South Africa; 3 University of Zimbabwe College of Health Sciences Clinical Trials Research Centre, Harare, Zimbabwe; 4 Makerere University-Johns Hopkins University (MU-JHU) Research Collaboration, Kampala, Uganda; 5 University of Malawi College of Medicine, Blantyre, Malawi; 6 UNC Project-Malawi, Lilongwe, Malawi; UNC-CH Department of Obstetrics and Gynecology, Chapel Hill, NC, United States of America; 7 Perinatal HIV Research Unit, University of the Witwatersrand, Johannesburg, South Africa; 8 Centre for the AIDS Programme of Research in South Africa (CAPRISA), Umlazi Clinical Research Site, Durban, South Africa; 9 Harvard T.H. Chan School of Public Health, Boston, MA, United States of America; 10 Johns Hopkins School of Medicine, Baltimore, MD, United States of America; The Ohio State University, UNITED STATES

## Abstract

**Background:**

The PROMOTE study aims to measure long-term antiretroviral treatment (ART) safety and adherence; compare HIV disease progression; assess subsequent adverse pregnancy outcomes; evaluate effect of ART exposure on growth and development in HIV-exposed uninfected children; and assess long-term survival of mothers and children. This report primarily describes cohort characteristics at baseline to better understand long-term outcomes.

**Methods and findings:**

This is a prospective study. HIV-infected mothers and their children originally recruited in a multisite randomized clinical trial for prevention of perinatal HIV transmission were re-enrolled in PROMOTE. A total of 1987 mothers and 1784 children were enrolled from eight sites in Uganda, Malawi, Zimbabwe and South Africa. Most women (≥75%) reported being married in Malawi and Zimbabwe compared to low proportions in South Africa (4.4% in Durban and 15% in Soweto), and 43.5% in Uganda (p<0.001). There were variabilities in contraceptive practices: injectable contraceptive was the commonest reported method (40.9% overall); implant was the second commonest (15.7% overall); oral contraceptives were common in Zimbabwe; and tubal ligation was common in Malawi and South Africa. At baseline, 97.8% of women reported currently using ART; 96.4% were in WHO clinical class 1 or 2; median CD4 cell count was 825 cells per uL; and viral load was undetectable in 1637 (~85%) of the women. Approximately, 14% of women did not inform their primary partners of their own HIV status, 18% reported that they knew their partners were not HIV tested, and 9% did not know if partner was tested. Overall mean age of children at enrollment was 3.5 years; and 5.7% and 25.0% had weight-for-age and height-for-age z-scores <2 standard deviations, respectively.

**Conclusions:**

These baseline data show high adherence to ART use. However, issues of HIV disclosure and reproductive intentions remain important. In addition to ART and ensuring high adherence, other preventive measures should be included.

## Introduction

The *Promoting Maternal and Infant Survival Everywhere* (PROMISE) multi-country randomized trial showed that perinatal HIV transmission can be reduced to ~0.5% when antiretroviral treatment (ART) is used during pregnancy and likewise post-partum continuation of maternal ART or infant prophylaxis can lead to very low rates of transmission during breastfeeding [1, 2]. Key agencies, including World Health Organization (WHO), now recommend use of lifelong ART regimens [3]. Based on the Population-based HIV Impact Assessment (PHIA) surveys it appears that several African countries are making progress in increasing number of individuals being HIV tested, initiating ART, and achieving high levels of viral suppression [4].

Therefore, major achievements have been made in reduction of HIV transmission, slower HIV disease progression and improved health as a result of viral suppression and improved immunological status. Nonetheless, the expanding use of ART across the life span requires long-term monitoring to assess safety and durability–especially among women and children as newer regimens are introduced. The importance of safety monitoring in reproductive age women is demonstrated by recent reports from Botswana where switching to dolutegravir has raised concerns of potential association of dolutegravir with adverse reproductive outcomes [5, 6]. In multiple settings in Sub-Saharan Africa, fertility remains high and subsequent pregnancies are common among HIV-infected women. Despite huge reductions in perinatal HIV transmission, the roll-out of ART has resulted in millions of HIV-exposed, uninfected infants and children, being exposed to approximately 30 months of ART. This includes both *in utero* from as early as conception through up to 18–24 months of breastfeeding during a period of rapid brain and somatic physical growth. Unfortunately, most ART drugs have limited research safety data on dosing and safety in pregnant or breastfeeding women and in children. This includes pregnancy outcomes and later pediatric growth and development [7]. WHO recommends toxicity and pregnancy outcome surveillance be conducted to provide safety information on ART regimens. This is particularly important in low-resource settings where malnutrition and other comorbidities are prevalent [8].

The *PEPFAR PROMise Ongoing Treatment Evaluation* (PROMOTE) study has been implemented to provide long-term follow-up data on safety outcomes of widespread use of ART among an already well-characterized cohort of HIV infected mothers and their children who previously enrolled in the multi-site PROMISE study [1]. The major specific aims of the PROMOTE study are to longitudinally evaluate adherence to ART and durability of consistent ART use among postpartum women; assess safety of long-term use of ART, including alternative regimens among women who initiated ART or switched use of ART during the PROMISE study from enrollment to end of follow-up postpartum; compare HIV disease progression by regimen and HIV-subtype; assess subsequent adverse pregnancy outcomes by regimen; and assess maternal and infant health outcomes to document the impact of long-term treatment. In this manuscript, we describe the PROMOTE study design, baseline data on women and children who enrolled in PROMOTE (originally enrolled in PROMISE), and discuss the significance of the study. These analyses primarily focus on baseline enrollment data to assist in understanding the long-term outcomes of the PROMOTE study.

## Materials and methods

### Design

**Fig 1:**
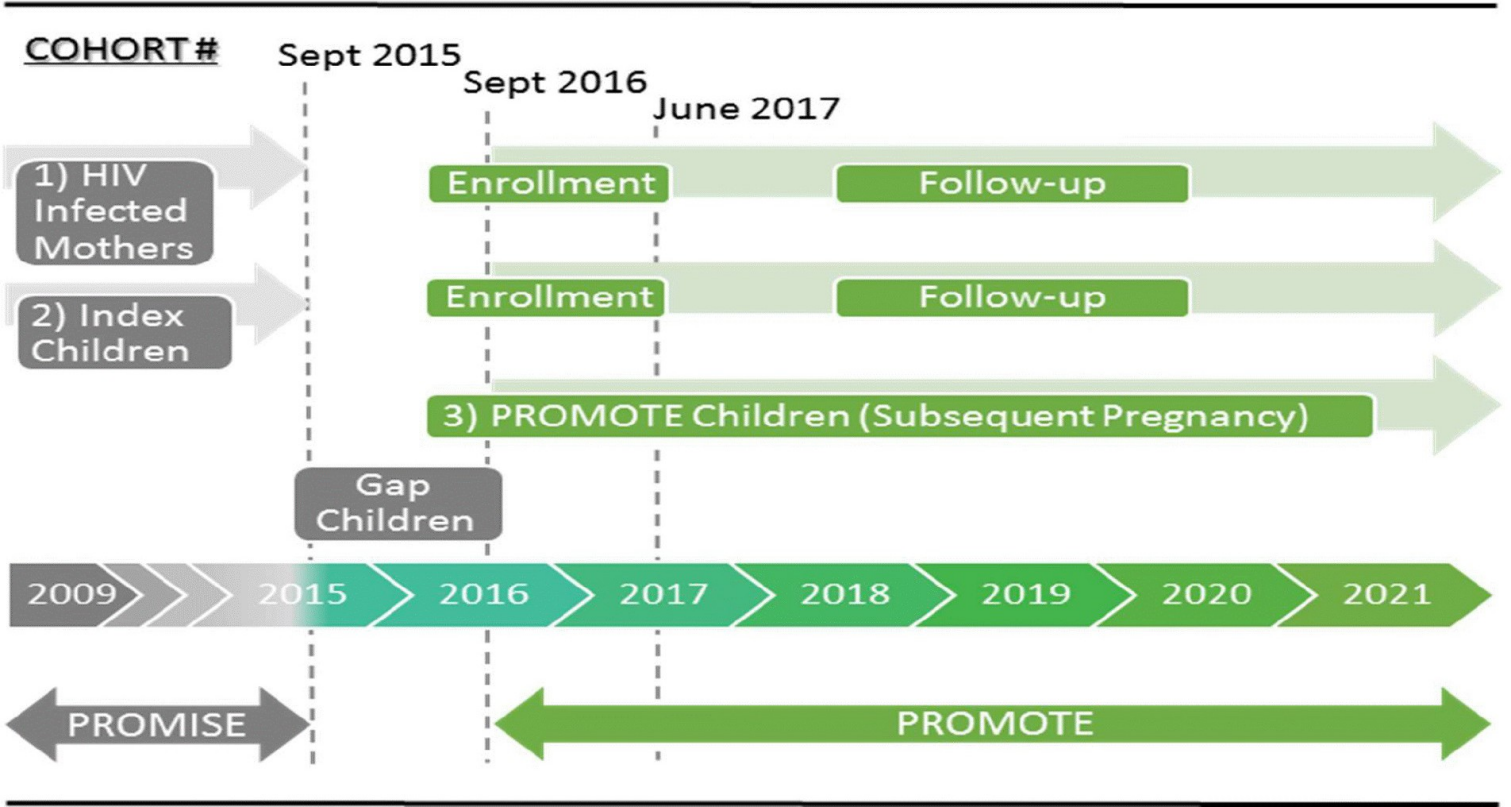
PROMOTE Study populations, enrollment and follow-up.

The PROMOTE study is an observational study to prospectively follow mothers and children who were originally recruited in the multi-country PROMISE randomized trial [1]. The PROMOTE enrollment started in December 2016—after termination and close-out of the PROMISE trial in 2015 (Fig 1). PROMOTE enrollment was completed in June 2017 and follow-up is planned to continue up to 2021.

### Setting and study populations

The PROMOTE study is conducted at eight research sites in four African countries: Makerere University Johns Hopkins University (MUJHU)/Kampala (Uganda), Blantyre and Lilongwe (Malawi), Harare, Seke North and St. Mary’s (Zimbabwe), Perinatal Health Research Unit (PHRU)/Johannesburg and uMlazi/Durban (South Africa). Fig 1 shows the study cohorts and the timing of their enrollment: 1) HIV-infected women who previously enrolled in the PROMISE trial from the eight African sites are enrolled and followed in the PROMOTE study; 2) The first born infants in PROMISE are enrolled in PROMOTE and followed with their mothers; 3) All children born during the PROMOTE follow-up as a result of a subsequent (repeat) pregnancy are enrolled and followed with their mothers. In addition to these distinct three cohorts, a small group of infants were born after closure of the PROMISE study and prior to start of the PROMOTE study. These “Gap” children were not formally enrolled; however, mothers were interviewed at PROMOTE baseline about the date of birth and survival status of these children.

### Inclusion criteria

Women and children enrolled in the PROMISE trial (and children born subsequently in PROMOTE) from one of eight African sites in Uganda, Malawi, Zimbabwe and South Africa; mothers willing to provide informed consent to enroll and continue follow-up in the PROMOTE study for herself and for her children); lives within the study catchment area; and has no plans to move during the study period. If a mother died, a caregiver can sign the informed consent to continue follow-up of the child who enrolled originally in PROMISE. If the child died, the mother can consent to be followed without her child.

### Exclusion criteria

Mother not able or willing to provide informed consent to continue follow-up in the PROMOTE study; plans to relocate permanently out of the catchment area during the cohort study period; judged by the site team and protocol chairs as having social or other reasons which would make it difficult for the mother/child pair to comply with study requirements.

### Study procedures for mothers

HIV-infected women previously enrolled in the PROMISE study were enrolled after appropriate counseling and consenting in the PROMOTE study. New study visits, schedule of evaluations, procedures, and study forms were implemented. The maternal screening and enrollment evaluations included: eligibility evaluations (e.g., obtaining written informed consent to participate in follow-up and collection of study samples), administration of sociodemographic questionnaires, medical history, physical examinations (e.g., WHO clinical staging and comorbid conditions such as TB), and provision of ART adherence counseling. ART is supplied per national guidelines as the prevailing standard of care in each country (efavirenz [EFV]-based regimen in Malawi, Zimbabwe and South Africa, and protease inhibitor [PI]-based regimen in Uganda). Enrollment laboratory evaluations included: complete blood count (CBC) with differential, CD4+ cell count, viral load, and serum chemistries (alanine aminotransferase, creatinine and creatinine clearance). Additional procedures included dual-energy X-ray absorptiometry (DXA scan), and storage of samples (blood samples for resistance testing and hair for cumulative drug levels). The maternal follow-up evaluations are conducted every six months and include: medical history, physical examination, adherence questionnaire (and counseling), and child neurodevelopmental questionnaire (every 12 months). Participants are allowed to attend interim visits for health conditions or issues pertaining to ART. Women who miss their scheduled visit during a certain window period or with abnormal laboratory results (e.g., high viral load) are actively followed at the location they provided and advised to return to the clinic for follow-up. Transportation costs are provided for follow-up visits. Data are collected at scheduled and interim visits as appropriate. The laboratory follow-up procedures were similar to the enrollment procedures. Women who enrolled in PROMOTE and subsequently had a repeat pregnancy followed a specific initial schedule that included pregnancy registration, confirmation of pregnancy, medical history and physical examination, and laboratory tests which included hepatitis B surface antigen test. Antenatal (monthly) and delivery evaluations and laboratory tests were also conducted similar to enrollment visits. At all scheduled and unscheduled visits evaluation of adverse events and classification was based on the DAIDS toxicity table (Version 2 [9]).

### Study procedures for children

Two cohorts of children are part of the PROMOTE study: those born during the PROMISE trial and those born subsequently during the PROMOTE study. The enrollment evaluations for Children born in the PROMISE trial included eligibility evaluations, clinical evaluations (medical history, physical examination [including neurological], documentation of the child’s HIV status, survival status and anthropometric measures). The enrollment evaluations also included laboratory tests (CBC with differential, HIV status), DXA scan, developmental testing, and Disability/ Child Behavior questionnaires. Follow-up evaluations for first born PROMISE and repeat pregnancy children included clinical and laboratory evaluations every 6 months.

### Data management

The PROMOTE study is managed by a Coordinating Center at the Johns Hopkins University in Baltimore, Maryland, and data management and statistical support is provided by a Data Management Center (DMC) at the Centre for the AIDS Programme of Research in South Africa (CAPRISA) in Durban, South Africa. PROMOTE study data are completed on designated case report forms (CRFs) by trained study workers. At the outset, research teams at all sites received training on the protocol, manual of operations, and quality control (QC)/quality assurance (QA) procedures. Regular monitoring of the study is provided by two designated scientists. Data QC/QA is conducted at the clinical sites, data are entered locally, and securely transferred to the DMC in South Africa. The DMC monitors site data entry of CRFs, data completion and provides refresher training whenever needed. A unique data sharing feature of the PROMOTE study is use of the same participant identification as in the PROMISE randomized clinical trial to allow data access from time of birth of the first child in PROMISE providing a follow-up opportunity of approximately 11 years for mothers and children from time of enrollment in PROMISE to completion of PROMOTE (this plan has been approved and included in the consent form).

### Statistical analyses

For the baseline data included in this report, we present descriptive and stratified analyses by research site and country. Current analyses are restricted to summaries of enrolled cohorts of women and children, and description of factors related to socioeconomic, reproductive, ART, and adverse events at the time of enrollment (baseline). Even though site specific data will be presented, data for few selected variables (marital status, socioeconomic status indicated by availability of water and electricity, travel time to the clinic, HIV status disclosure and CD4 cell count) were included by country for statistical testing purposes. Fisher-Freeman-Halton test and Kruskal-Wallis test were used to determine if there was an association between countries and few selected categorical and continuous variables, respectively.

### Study approval and provision of clinical care

The PROMOTE study was approved by all relevant institutional review boards (IRBs) in the U.S. and collaborating African research sites/countries. These are: MUJHU/Kampala, Uganda: The Joint Clinical Research Centre (JCRC) IRB in Uganda and The Johns Hopkins Medical Institutions (JHMI) IRB in the U.S.; Blantyre, Malawi: College of Medicine Research and Ethics Committee (COMREC) in Malawi and Johns Hopkins Medical Institutions (JHMI) IRB in the U.S.; Lilongwe, Malawi: National Health Sciences Research Committee (NHSRC) in Malawi and University of North Carolina, Chapel Hill (UNC-CH) Office of Human Research Ethics IRB in the U.S.; Harare, Seke North and St. Mary’s sites, Zimbabwe: Medical Research Council of Zimbabwe (MRCZ) National Ethics Committee; PHRU, Johannesburg, South Africa: University of Witwatersrand Human Research Ethics Committee (Medical); and uMlazi, Durban, South Africa: Biomedical Research Ethics Committee and Kwazulu-Natal Department of Health. All women signed a written informed consent form to enroll and be followed with their children for the duration of the study and to provide study samples. All women are on ART regimens provided as the standard of care in each country. The PROMOTE study provides at no cost laboratory testing and clinical management as necessary. Children found HIV-infected are started on ART per country-specific regimens and guidelines. Maternal counseling on antiretroviral adherence and other health issues are also provided at the clinics.

## Results

Overall, 1987 mothers and 1784 children were enrolled in PROMOTE from the PROMISE study (Table 1). Additionally, 134 children were born and enrolled during PROMOTE follow-up for a total of 3905 women and children (Table 1). Approximately, 340 children not first born in PROMISE (“Gap” children) were born to PROMISE mothers before entry into the PROMOTE study and not formally enrolled. The number of new pregnancies in PROMOTE to the time of this analysis and the outcomes of these pregnancies are also shown in Table 1.

**Table 1:** PROMOTE enrollment by country and research site.

Country	Uganda	Malawi		Zimbabwe			South Africa		
Research Site	MUJHU-Kampala	Blantyre	Lilongwe	Harare	Seke North	St. Mary’s	PHRU-Soweto	uMlazi-Durban	Total
Cohort 1: HIV-infected mothers	352	343	322	94	179	175	273	249	1987
Cohort 2: First born children in PROMISE	310	317	289	91	169	162	244	202	1784
Cohort 3: PROMOTE children (subsequent pregnancies at time of analysis)	27	35	24	4	13	13	15	3	134
*All children*	***337***	***352***	***313***	***95***	***182***	***175***	***259***	***205***	***1918***
**Total mothers and children enrolled**	**689**	**695**	**635**	**189**	**361**	**350**	**532**	**454**	**3905**
**PROMOTE pregnancies**									
New pregnancies at time of analyses	64	49	45	11	29	29	42	15	**284**
Number with pregnancy outcomes	27	31	24	4	14	14	19	3	**136**
Pregnancy outcomes, n (%)									
Live birth	24 (88.9%)	30 (96.8%)	20 (83.3%)	2 (50.0%)	13 (92.9%)	14 (100.0%)	15 (78.9%)	3 (100.0%)	**121 (89.0%)**
Low birth weight <2500g	1 (3.7%)	0	2 (8.3%)	1 (25.0%)	0	0	1 (5.3%)	0	**5 (3.7%)**
Preterm delivery <37 weeks	1 (3.7%)	0	1 (4.2%)	0	0	0	0	0	**2 (1.5%)**
Stillbirth/Intrauterine fetal death >_20 weeks	0	1 (3.2%)	0	0	1 (7.1%)	0	1 (5.3%)	0	**3 (2.2%)**
Abortion/miscarriage <20weeks	0	0	1 (4.2%)	1 (25.0%)	0	0	2 (10.5%)	0	**4 (2.9%)**
Congenital anomalies	1 (3.7%)	0	0	0	0	0	0	0	**1 (0.7%)**

Selected baseline characteristics of the 1784 children enrolled in PROMOTE from the PROMISE study are shown in Table 2. The overall mean age was 3.5 years. The overall proportions of children with weight-for-age and height-for-age z-scores <2 standard deviations (SDs) were 5.7% and 25.0%, respectively (with wide variabilities across sites).

**Table 2:** Baseline characteristics of children enrollment in PROMOTE study.

	Uganda	Malawi		Zimbabwe			South Africa		
	MUJHU-Kampala (N = 310)	Blantyre (N = 317)	Lilongwe (N = 289)	Harare (N = 91)	Seke North (N = 169)	St. Mary’s (N = 162)	PHRU-Soweto (N = 244)	uMlazi-Durban (N = 202)	Total (N = 1784)
First born children in PROMISE									
Gender, n (%) (N = 1781)									
Male	161 (52.1%)	164 (51.7%)	156 (54.0%)	41 (45.1%)	88 (52.1%)	84 (51.9%)	123 (50.6%)	98 (48.8%)	915 (51.4%)
Female	148 (47.9%)	153 (48.3%)	133 (46.0%)	50 (54.9%)	81 (47.9%)	78 (48.1%)	120 (49.4%)	103 (51.2%)	866 (48.6%)
Children age–years, Mean (SD)*	3.6 (1.0)	3.3 (1.0)	3.7 (0.9)	3.6 (1.1)	3.6 (0.9)	3.4 (1.1)	3.5 (1.0)	3.7 (1.0)	3.5 (1.0)
Anthropometrics									
Weight-for-age (N = 1775)									
Mean z-score	-0.50	-0.91	-0.71	-0.71	-0.43	-0.51	-0.25	-0.08	-0.53
SD* for z-score	0.86	0.87	0.97	0.88	0.87	0.94	1.09	0.96	0.97
Proportion below 3 SD*	1 (0.3%)	6 (1.9%)	3 (1.0%)	1 (1.1%)	1 (0.6%)	0	1 (0.4%)	0	13 (0.7%)
Proportion below 2 SD*	10 (3.2%)	30 (9.5%)	28 (9.8%)	6 (6.6%)	3 (1.8%)	9 (5.6%)	11 (4.5%)	5 (2.5%)	102 (5.7%)
Length/height-for-age (N = 1769)									
Mean z-score	-1.16	-2.00	-1.72	-0.79	-1.29	-1.17	-0.69	-0.84	-1.29
SD* for z-score	0.98	1.18	1.01	1.00	1.17	0.96	1.34	1.12	1.20
Proportion below 3 SD*	7 (2.3%)	54 (17.4%)	26 (9.0%)	1 (1.1%)	12 (7.1%)	6 (3.7%)	7 (2.9%)	5 (2.5%)	118 (6.7%)
Proportion below 2 SD*	60 (19.5%)	137 (44.2%)	98 (33.9%)	12 (13.2%)	43 (25.4%)	30 (18.5%)	33 (13.8%)	29 (14.4%)	442 (25.0%)

*SD: Standard deviation

Table 3 shows maternal sociodemographic characteristics by site and country. Most of the characteristics were comparable across sites. In particular, there were substantial and statistically significant differences among countries by marital status (p<0.001), socioeconomic status (indicated by availability of electricity (p<0.001) and water in the living premises (p<0.001)), and travel time to the study clinic (p<0.001). For example, most women (75% or more) reported being married in Malawi and Zimbabwe compared to very low rates in South Africa (4.4% in Durban and 15% in Soweto), and 43.5% in Uganda. Although overall, 30.4% of the women reported 1 hour or more of travel time to access the specific study clinic, there was a wide variability– 58.0% in Uganda, 36.6%-39.9% in Malawi, and much less in Zimbabwe and South Africa. Table 3 also shows maternal nutritional status based on body mass index (BMI) assessment (kg/m^2^). The overall frequency of being underweight (BMI <18.5) was 4.1%; the sites in Blantyre, Malawi and Seke North, Zimbabwe showed highest frequencies (9.0% and 6.1%, respectively).

**Table 3:** Maternal baseline sociodemographic characteristics by country and research site.

	Uganda	Malawi		Zimbabwe			South Africa		
Variable	MUJHU-Kampala (N = 352)	Blantyre (N = 343)	Lilongwe (N = 322)	Harare (N = 94)	Seke North (N = 179)	St. Mary’s (N = 175)	PHRU-Soweto (N = 273)	uMlazi-Durban (N = 249)	Total (N = 1987)
Age–years, Mean (SD)	30.7 (5.4)	30.7 (4.9)	31.2 (5.4)	32.3 (6.2)	32.2 (5.2)	31.6 (5.1)	32.9 (5.5)	30.5 (5.2)	31.4 (5.4)
Marital status, n (%)^*^									
No regular partner**	38 (10.8%)	2 (0.6%)	5 (1.6%)	2 (2.1%)	4 (2.2%)	8 (4.6%)	62 (22.7%)	41 (16.5%)	162 (8.2%)
Primary regular partner**	114 (32.4%)	4 (1.2%)	16 (5.0%)	4 (4.3%)	14 (7.8%)	12 (6.9%)	167 (61.2%)	196 (78.7%)	527 (26.5%)
Married	153 (43.5%)	263 (76.7%)	248 (77.0%)	74 (78.7%)	140 (78.2%)	131 (74.9%)	41 (15.0%)	11 (4.4%)	1061 (53.4%)
Separated	39 (11.1%)	20 (5.8%)	16 (5.0%)	5 (5.3%)	5 (2.8%)	10 (5.7%)	1 (0.4%)	1 (0.4%)	97 (4.9%)
Divorced	0	27 (7.9%)	24 (7.5%)	3 (3.2%)	4 (2.2%)	3 (1.7%)	0	0	61 (3.1%)
Widowed	8 (2.3%)	27 (7.9%)	13 (4.0%)	6 (6.4%)	12 (6.7%)	11 (6.3%)	2 (0.7%)	0	79 (4.0%)
Electricity in the premises, n (%)^*^	275 (78.1%)	192 (56.0%)	89 (27.6%)	55 (58.5%)	122 (68.2%)	113 (64.6%)	264 (96.7%)	242 (97.2%)	1352 (68.0%)
Tap water in the premises, n (%)^*^	149 (42.3%)	244 (71.1%)	137 (42.5%)	55 (58.5%)	114 (63.7%)	107 (61.1%)	272 (99.6%)	224 (90.0%)	1302 (65.5%)
Travel time from home to clinic, n (%) ^a^^*^									
Less than 30 minutes	19 (5.4%)	43 (12.5%)	22 (6.9%)	42 (44.7%)	107 (59.8%)	69 (39.4%)	100 (36.8%)	94 (37.8%)	496 (25.0%)
30–60 minutes	129 (36.6%)	175 (51.0%)	171 (53.3%)	37 (39.4%)	51 (28.5%)	70 (40.0%)	137 (50.4%)	116 (46.6%)	886 (44.6%)
1–2 hours	136 (38.6%)	101 (29.4%)	101 (31.5%)	14 (14.9%)	17 (9.5%)	27 (15.4%)	32 (11.8%)	31 (12.4%)	459 (23.1%)
Greater than 2 hours	68 (19.3%)	24 (7.0%)	27 (8.4%)	1 (1.1%)	4 (2.2%)	9 (5.1%)	3 (1.1%)	8 (3.2%)	144 (7.3%)
Body mass index (kg/m^2^)									
<18.5	8 (2.3%)	31 (9.0%)	10 (3.1%)	3 (3.2%)	11 (6.1%)	6 (3.4%)	7 (2.6%)	5 (2.0%)	81 (4.1%)
18.5–24.9	164 (46.6%)	194 (56.6%)	190 (59.0%)	49 (52.1%)	80 (44.7%)	98 (56.0%)	73 (26.7%)	81 (32.5%)	929 (46.8%)
25.0–29.9	93 (26.4%)	81 (23.6%)	72 (22.4%)	18 (19.1%)	49 (27.4%)	46 (26.3%)	85 (31.1%)	60 (24.1%)	504 (25.4%)
≥30.0	87 (24.7%)	37 (10.8%)	50 (15.5%)	24 (25.5%)	39 (21.8%)	25 (14.3%)	108 (39.6%)	103 (41.4%)	473 (23.8%)

^**a**^2 participants had missing data;

* p-value<0.001 calculated from Fisher-Freeman-Halton test;

** No regular partner: indicates casual partner and Primary regular partner indicates the main co-inhabiting individual identified by the respondent. There were also differences by site and country for reported reproductive history and contraceptive use as shown in Table 4. Overall, the injectable contraceptive was the commonest method used (40.9%)–especially at the two South African sites (>55%). The implant was the second commonest method used (15.7%). However, there were variations: the oral contraceptive was popular at the Zimbabwe sites (40.2%-54.7%) and tubal ligation was common at the Malawi (7.5%-12.9%) and South Africa (7.3%-10.3%) sites. Other than in Uganda, ~50% or more women reported did not “want to have another child”. The frequency of unplanned immediate prior pregnancy was 29.3% overall–but very high at the uMlazi/Durban site (52.2%) and low at the Uganda site (9.9%); relatively high at the Malawi sites (>30%).

**Table 4:** Maternal reproductive history by country and research site.

	Uganda	Malawi		Zimbabwe			South Africa		
Variable	MUJHU-Kampala (N = 352)	Blantyre (N = 343)	Lilongwe (N = 322)	Harare (N = 94)	Seke North (N = 179)	St. Mary’s (N = 175)	PHRU-Soweto (N = 273)	uMlazi-Durban (N = 249)	Total (N = 1987)
Had sex in the last 3 months, n (%)	257 (73.0%)	249 (72.6%)	227 (70.5%)	74 (78.7%)	148 (82.7%)	140 (80.0%)	187 (68.5%)	183 (73.5%)	1465 (73.7%)
Condoms use, n (%) ^b^									
Always	33 (12.8%)	57 (22.9%)	71 (31.3%)	36 (48.6%)	101 (68.2%)	97 (69.3%)	90 (48.4%)	84 (45.9%)	569 (38.9%)
Sometimes	66 (25.7%)	109 (43.8%)	143 (63.0%)	29 (39.2%)	43 (29.1%)	36 (25.7%)	67 (36.0%)	89 (48.6%)	582 (39.8%)
Never	158 (61.5%)	83 (33.3%)	13 (5.7%)	9 (12.2%)	4 (2.7%)	7 (5.0%)	29 (15.6%)	10 (5.5%)	313 (21.4%)
Used family planning methods, n (%)	169 (74.1%)	194 (85.1%)	199 (93.9%)	53 (74.6%)	127 (92.7%)	115 (91.3%)	138 (78.9%)	174 (97.2%)	1169 (86.2%)
Family planning contraceptive method used (if woman reported sex during past 3 months at baseline)^e^									
Injectables	68 (40.5%)	78 (40.2%)	92 (46.2%)	9 (17.0%)	21 (16.5%)	33 (28.7%)	76 (55.5%)	100 (57.5%)	477 (40.9%)
Condoms	50 (29.8%)	43 (22.2%)	43 (21.6%)	3 (5.7%)	13 (10.2%)	18 (15.7%)	23 (16.8%)	29 (16.7%)	222 (19.0%)
Implant	20 (11.9%)	37 (19.1%)	40 (20.1%)	10 (18.9%)	39 (30.7%)	12 (10.4%)	3 (2.2%)	22 (12.6%)	183 (15.7%)
Oral	19 (11.3%)	2 (1.0%)	4 (2.0%)	29 (54.7%)	51 (40.2%)	51 (44.3%)	21 (15.3%)	3 (1.7%)	180 (15.4%)
Tubal ligation	4 (2.4%)	25 (12.9%)	15 (7.5%)	0	1 (0.8%)	0	10 (7.3%)	18 (10.3%)	73 (6.3%)
Intrauterine contraceptive device	5 (3.0%)	5 (2.6%)	3 (1.5%)	2 (3.8%)	2 (1.6%)	1 (0.9%)	3 (2.2%)	1 (0.6%)	22 (1.9%)
Withdrawal, herbs or emergency pill	2 (1.2%)	4 (2.1%)	0	0	0	0	0	0	6 (0.5%)
Hysterectomy	0	0	2 (1.0%)	0	0	0	1 (0.7%)	1 (0.6%)	4 (0.3%)
Currently breastfeeding, n (%)^c^	30 (8.6%)	41 (12.0%)	49 (15.3%)	15 (16.1%)	26 (14.5%)	26 (14.9%)	5 (1.8%)	3 (1.2%)	195 (9.8%)
Family size desired from the outset, median(IQR)[min-max]	4 (4–6) [1–18]	3 (3–4) [1–7]	3 (3–4) [0–8]	4 (3–4) [0–10]	4 (3–4) [0–6]	4 (3–4) [1–7]	3 (2–4) [0–7]	3 (2–4) [0–10]	4 (2–4) [0–18]
Want to have another child, n (%)^d^									
Yes	224 (63.6%)	100 (29.2%)	126 (39.1%)	41 (43.6%)	72 (40.2%)	70 (40.0%)	83 (30.5%)	86 (34.5%)	802 (40.4%)
No	111 (31.5%)	223 (65.0%)	184 (57.1%)	44 (46.8%)	99 (55.3%)	89 (50.9%)	180 (66.2%)	136 (54.6%)	1066 (53.7%)
Not sure	17 (4.8%)	20 (5.8%)	12 (3.7%)	9 (9.6%)	8 (4.5%)	16 (9.1%)	9 (3.3%)	27 (10.8%)	118 (5.9%)
How many more children would like to have, median (IQR)	1 (1–2)	1 (1–2)	1 (1–1)	1 (1–2)	1 (1–2)	1 (1–2)	1 (1–2)	1 (1–2)	1 (1–2)
Was the last pregnancy planned?, n (%)^e^									
Wanted to be pregnant	251 (71.3%)	167 (48.7%)	137 (42.5%)	66 (70.2%)	104 (58.1%)	104 (59.4%)	120 (44.3%)	45 (18.1%)	994 (50.1%)
Wanted to wait until sometime later	66 (18.8%)	58 (16.9%)	62 (19.3%)	12 (12.8%)	40 (22.3%)	25 (14.3%)	72 (26.6%)	74 (29.7%)	409 (20.6%)
Did not want to be pregnant	35 (9.9%)	118 (34.4%)	123 (38.2%)	16 (17.0%)	35 (19.6%)	46 (26.3%)	79 (29.2%)	130 (52.2%)	582 (29.3%)
Total pregnancies including current, median (IQR)	4 (3–5)	3 (3–4)	4 (2–5)	3 (2–4)	3 (2–4)	3 (2–4)	3 (2–4)	3 (2–3)	3 (2–4)
Number children alive today, median (IQR)	3 (2–4)	3 (2–4)	3 (2–4)	3 (2–3)	3 (2–3	2 (2–3)	2 (1–3)	2 (2–3)	3 (2–3)

^**b**^1 with missing data,

^c^7 with missing data,

^d^1 with missing data,

Table 5 shows maternal baseline ART information by research site and country. Overall, 97.8% of women reported currently taking ART at enrollment in PROMOTE study; the rates were uniformly high at all sites and only 43 (2.2%) women were not using ART. Few (3.5%) women were in WHO clinical class 3 or 4. The overall median CD4 cell count at baseline was high: 825.5 cells per uL with minimal variability between sites within the same country. However, the differences in CD4 count (cells per uL) among countries were statistically significant (Uganda: 963.5; Malawi: 739; Zimbabwe: 911.5; South Africa: 794; p<0.001). The viral load (VL) detectability using various cutoff points is shown in Table 5. Overall, VL was not detectable in 1628 (~85%) of the women. Of the 292 women with detectable VL, in 77 women VL was ≤200 copies/mL, in 17 women VL was 200–399 copies/mL, in 32 women VL was 400–1000 copies/mL and in 166 VL was >1000 copies/mL. Table 5 also shows that overall, ~14% of women have not told their partners of their own HIV status–this was especially high in Uganda (38.6%) and at the two South African sites (20.2% in Soweto and 21.3% in Durban). The differences in this characteristic across countries were statistically significant (Uganda: 38.6%; Malawi: 3.8%; Zimbabwe: 3.2%; South Africa 20.7%; (p<0.001). Additionally, 72.8% of women reported that they knew their primary partners were HIV tested; 18.1% reported partner was not HIV tested; and 9.1% reported not knowing if partner was HIV tested.

**Table 5:** Maternal baseline antiretroviral treatment information by country and research site.

	Uganda	Malawi		Zimbabwe			South Africa		
Variable	MUJHU-Kampala (N = 352)	Blantyre (N = 343)	Lilongwe (N = 322)	Harare (N = 94)	Seke North (N = 179)	St. Mary’s (N = 175)	PHRU-Soweto (N = 273)	uMlazi-Durban (N = 249)	Total (N = 1987)
Currently taking ARV treatment, n (%) ***	348 (98.9%)	335 (97.7%)	308 (95.7%)	91 (96.8%)	177 (98.9%)	174 (99.4%)	264 (96.7%)	247 (99.2%)	1944 (97.8%)
WHO clinical classification, n (%)^f^									
1	239 (69.1%)	319 (93.3%)	302 (94.4%)	84 (90.3%)	137 (76.5%)	159 (91.9%)	261 (99.6%)	211 (86.1%)	1712 (87.3%)
2	88 (25.4%)	11 (3.2%)	10 (3.1%)	8 (8.6%)	29 (16.2%)	11 (6.4%)	0	22 (9.0%)	179 (9.1%)
3	16 (4.6%)	8 (2.3%)	7 (2.2%)	1 (1.1%)	12 (6.7%)	3 (1.7%)	0	10 (4.1%)	57 (2.9%)
4	3 (0.9%)	4 (1.2%)	1 (0.3%)	0	1 (0.6%)	0	1 (0.4%)	2 (0.8%)	12 (0.6%)
Viral load, copies/mL, n (%)^g^									
>1000	25 (7.1%)	38 (12.5%)	52 (16.3%)	6 (6.4%)	8 (4.5%)	17 (9.7%)	8 (3.0%)	12 (5.2%)	166 (8.6%)
400–1000	4 (1.1%)	6 (2.0%)	9 (2.8%)	3 (3.2%)	1 (0.6%)	3 (1.7%)	3 (1.1%)	3 (1.3%)	32 (1.7%)
200–399	4 (1.1%)	2 (0.7%)	3 (0.9%)	1 (1.1%)	3 (1.7%)	0	1 (0.4%)	3 (1.3%)	17 (0.9%)
<200	28 (8.0%)	8 (2.6%)	10 (3.1%)	4 (4.3%)	5 (2.8%)	4 (2.3%)	8 (3.0%)	10 (4.3%)	77 (4.0%)
Undetectable	291 (82.7%)	251 (82.3%)	246 (76.9%)	80 (85.1%)	162 (90.5%)	151 (86.3%)	251 (92.6%)	205 (88.0%)	1637 (84.9%)
CD4 count (cells/uL), median (IQR)**	963.5 (747 – 1189.5)	764 (593 – 965)	727 (545 – 904)	959.5 (779–1170)	928 (761–1069)	858 (646–1081)	727 (601 – 880)	886 (721.5–1083.5)	825 (647 – 1035)
Disclosed HIV status to primary partner, n (%) †	164 (61.4%)	253 (94.8%)	258 (97.7%)	77 (98.7%)	148 (96.1%)	138 (96.5%)	166 (79.8%)	163 (78.7%)	1367 (86.1%)
Has partner been tested for HIV, n (%)†									
Yes	155 (58.1%)	203 (76.0%)	213 (80.7%)	66 (84.6%)	124 (80.5%)	115 (80.4%)	146 (70.2%)	134 (64.7%)	1156 (72.8%)
No	23 (8.6%)	61 (22.8%)	42 (15.9%)	10 (12.8%)	27 (17.5%)	24 (16.8%)	45 (21.6%)	56 (27.1%)	288 (18.1%)
Don’t know	89 (33.3%)	3 (1.1%)	9 (3.4%)	2 (2.6%)	3 (1.9%)	4 (2.8%)	17 (8.2%)	17 (8.2%)	144 (9.1%)

^f^27 missing data,

^g^ 58 missing data,

† among 1588 who had partners,

*p-value<0.001 calculated from Fisher-Freeman-Halton test;

**p-value <0.001 from Kruskal-Wallis test,

*** 85.0% on efavirenz-based regimen (>95% at all sites >95% other than MUJHU, Kampala site (~30%)) and 15.0% on protease inhibitor-based regimen (all site <5% other than MUJHU, Kampala site (69%)).

## Discussion

The PROMOTE cohort represents a hybrid example where participants in a highly organized clinical trial setting are transitioned to a standard of care setting which includes provision of country-specific ART regimens and clinical care. Therefore, it provides a unique opportunity to evaluate and assess clinical outcomes which is not possible through country-level service statistics. Women who enrolled in the PROMOTE study have been receiving counseling and care for almost five years in a structured clinical setting during the PROMISE trial. Analyses of the socio-demographic, reproductive and ART findings at enrollment by site and country provide immediate and important information to assess HIV treatment and prevention in women. Future studies in PROMOTE will elucidate treatment effects beyond the PROMISE pregnancy and early postpartum. The PROMOTE data are needed to understand the durability and safety of universal ART and monitor trends in behaviors such as fertility decisions after prolonged use of ART [10, 11].

The overall achievements relating to ART use are encouraging and consistent with what was observed earlier in this cohort of healthy women for prevention of HIV transmission during pregnancy and postpartum (~0.5% perinatal transmission [1, 2]). The PROMOTE baseline data show that 98% of the women are continuing to use ART and ~85% had undetectable virus in blood samples (with high CD4 cell counts and lack of advanced HIV clinical disease) suggesting high adherence. These findings are consistent with data from the PHIA surveys conducted in Malawi, Zimbabwe and Zambia where 87% who knew their HIV status were on ART and 89% had viral suppression below 1000 copies/mL (4) as well as earlier data from other African settings [12,13].

Nonetheless, few baseline findings from this study raise concerns. For example, despite years of counseling on HIV during the PROMISE study, 14% of the women (higher in Uganda and South African sites) did not inform their primary partners about their HIV status, and 9% of the women reported not knowing if their partners were HIV tested. Some of the socioeconomic and reproductive characteristics of this cohort of HIV-infected women may explain these inadequacies–and highlight the difficulties that health care providers are facing in these countries. Although variable across site, there were substantial differences in marital status; only 53% of women reported being married—these were mostly in Malawi and Zimbabwe. The frequency of not having regular partnership was high in the South African sites and Uganda. The frequency of nonuse of contraception methods was also high at some sites. A more striking finding in this baseline evaluation (and a prominent emerging concern during the on-going follow-up) is the high frequency of subsequent pregnancies–suggesting changes in fertility whether desired or unplanned. At some sites, more than 40% of women wanted to be pregnant and the frequency of unwanted pregnancy was high. These same factors have been well-described in early studies of HIV infection in sub-Saharan Africa [14–18]. What is currently emerging is likely the effect of ART and the new expectation and desires of these women. It is expected that consistent use of ART will minimize HIV transmission. However, repeat pregnancies at short intervals can impact infant and maternal health independent of HIV and ART use [19]. In the era of universal ART use, strategies that maximize maternal health should be addressed at the individual, couples and health care provider levels.

A major strength of this study is the large sample size obtained from multiple sites and countries allowing for generalizability of the findings. Although current data are only from the baseline enrollment visit, it should be realized that these women and children have been under follow-up for several years. Therefore, assessment of previous interventions such as maintenance of ART adherence and counseling on HIV prevention will be examined in future studies. A potential limitation of the current analysis is its cross-sectional nature; however, future analyses will build on these baseline findings and provide longitudinal assessments.

Despite the global successes in prevention and treatment of HIV and acknowledging variability between sites and countries, these data from sub-Saharan Africa reveal some important policy challenges. Better approaches are needed to address and manage some chronic constraints such as gender power imbalances and inadequate socio-economic situations [20–23]. Additionally, more work is needed in some essential prevention approaches. ART is well accepted and there are definite improvements in maternal health—but how to overcome the barriers that are entangled with cultural and societal norms such as the desire for more children and the continued high fertility? The success of ART should provide a venue to address the reproductive health needs of these women; e.g., emphasis on the appropriate spacing of the inter-pregnancy interval through use and making available safe contraceptive approaches. Building on these baseline data, future PROMOTE reports will focus on long-term safety, adherence to different ART regimens, progression of HIV disease and survival of mothers and children.

## Disclaimer

The findings and conclusions reported herein are those of the author(s) and do not necessarily reflect the official position of the U.S. government.

## References

[1] Fowler MG, Qin M, Fiscus S, Currier J, Flynn P, Chipato T, et al Benefits and risks of antiretroviral therapy for perinatal HIV prevention. N Engl J Med 2016; 375:1726–1737. 10.1056/NEJMoa1511691 27806243 10.1056/NEJMoa1511691 PMC5214343

[2] Flynn P, Taha TE, Cababasay M, Fowler MG, Mofenson LM, Owor M, et al Prevention of HIV-1 transmission through breastfeeding: Efficacy and safety of maternal antiretroviral versus infant nevirapine prophylaxis for duration of breastfeeding in HIV-1-infected women with high CD4 cell count (IMPAACT PROMISE): a randomized, open label, clinical trial. J Acquir Immune Defic Syndr 2018; 77:383–392. 10.1097/QAI.0000000000001612 29239901 10.1097/QAI.0000000000001612 PMC5825265

[3] World Health Organization. Consolidated Guidelines on the Use of Antiretroviral Drugs for Treating and Preventing HIV Infection. 2016; Geneva: World Health Organization. 27466667

[4] Population-based HIV Impact Assessment (PHIA). http://phia.icap.columbia.edu/; accessed April 2, 2018.

[5] WHO. Potential safety issue affecting women living with HIV using dolutegravir at time of conception. 5 18, 2018 https://penta-id.org/news/presentation/who-and-ema-statements-on-potential-safety-issue-affecting-women-living-with-hiv-using-dolutegravir/ (accessed June 25, 2018).

[6] PEPFAR statement on potential safety issue affecting women living with HIV using dolutegravir at time of conception. May 18, 2018.

[7] Mofenson LM. Antiretroviral therapy and adverse pregnancy outcome: The elephant in the room? J Infect Dis, 2016; 213: 1051–54. 10.1093/infdis/jiv390 26265779 10.1093/infdis/jiv390

[8] Newell ML, Bunders MJ. Safety of antiretroviral drugs in pregnancy and breastfeeding for mother and child. Curr Opin HIV AIDS, 2013;8:504–510. 10.1097/COH.0b013e3283632b88 23743789 10.1097/COH.0b013e3283632b88

[9] Division of AIDS (DAIDS) Table for Grading the Severity of Adult and Pediatric Adverse Events. Version 2, 2014.

[10] Jaquet A, Djima MM, Coffie P, Die Kacou H, Eholie SP, Messou E, et al Pharmacovigilance for antiretroviral drugs in Africa, lessons from a study in Abidjan, Cote d’Ivoire: Pharmacovigilance for antiretroviral drugs in Africa. Pharmacoepidemiol Drug Saf, 2011;20: 1303–1310. 10.1002/pds.2182 21735508 10.1002/pds.2182 PMC3227553

[11] Yeatman S, Eaton JW, Beckles Z, Benton L, Gregson S, Zaba B. Impact of ART on the fertility of HIV-positive women in sub-Saharan Africa. Trop Med Int Health, 2016;21:1071–1085. 10.1111/tmi.12747 27371942 10.1111/tmi.12747

[12] Barbara CB, Kiragga A, Mubiru F, Kambugu A, Kamya M, Reynolds SJ. First‐line antiretroviral therapy durability in a 10‐year cohort of naïve adults started on treatment in Uganda. J Int AIDS Soc, 2016;19:20773 http://www.jiasociety.org/index.php/jias/article/view/20773 | 10.7448/IAS.19.1.20773 (accessed April 4, 2018). 27319742 PMC4913145

[13] Billioux A, Nakigozi G, Newell K, Chang LW, Quinn TC, Gray RH, et al Durable Suppression of HIV-1 after Virologic MonitoringBased Antiretroviral Adherence Counseling in Rakai, Uganda. PLoS ONE 2015;10: e0127235 10.1371/journal.pone.0127235 26011158 10.1371/journal.pone.0127235 PMC4444255

[14] Lurie MN, Williams BG, Zuma K, Mkaya-Mwamburi D, Garnett GP, Sturm AW, et al The impact of migration on HIV-1 transmission in South Africa: A study of migrant and nonmigrant men and their partners. Sex Transm Dis 2003; 30: 149–156. 12567174 10.1097/00007435-200302000-00011

[15] Kharsany ABM, Karim QA. HIV infection and AIDS in sub-Saharan Africa: current status, challenges and opportunities. The Open AIDS J, 2016;10:34–48. 10.2174/1874613601610010034 27347270 10.2174/1874613601610010034 PMC4893541

[16] Cherutich P, Kaiser R, Galbraith J, Williamson J, Shiraishi RW, Ngare C, et al Lack of Knowledge of HIV Status a Major Barrier to HIV Prevention, Care and Treatment Efforts in Kenya: Results from a Nationally Representative Study. PLoS ONE, 2012; 7: e36797 10.1371/journal.pone.0036797 22574226 10.1371/journal.pone.0036797 PMC3344943

[17] Marston M, Zaba B, Eaton JW. The relationship between HIV and fertility in the area of antiretroviral therapy in sub-saharan Africa: evidence from 49 Demographic and Health Surveys. Trop Med Int Health 2017;22:1542–1550. 10.1111/tmi.12983 28986949 10.1111/tmi.12983 PMC5716842

[18] Holmes CB, Yiannoutsos CT, Elul B, Bukusi E, Ssali J, Kambugu A, et al Increased prevalence of pregnancy and comparative risk of program attrition among individuals starting HIV treatment in East Africa. PLoS ONE, 2018; 13: e0190828 10.1371/journal.pone.0190828 Accessed April 4, 2018. 29342180 PMC5771608

[19] Kozuki N, Lee AC, Silveira MF, Victora CG, Adair L, Humphrey J, et al The association of birth intervals with small-for-gestational age, preterm, and neonatal and infant mortality: a meta-analysis. BMC Public Health, 2013;13 Suppl 3:S3. 10.1186/1471-2458-13-S3-S3 PMC3847557 24564484

[20] Langen TT. Gender power imbalances on women’s capacity to negotiate self-protection against HIV/AIDS in Botswana and South Africa. Afr Health Sci, 2005;5:188–197. 10.5555/afhs.2005.5.3.188 16245988 10.5555/afhs.2005.5.3.188 PMC1831928

[21] Ramjee G, Daniels B. Women and HIV in sub-Saharan Africa. AIDS Res Ther 2013;10:30 http://www.aidsrestherapy.com/content/10/1/30. Accessed April 4, 2018. 10.1186/1742-6405-10-30 24330537 10.1186/1742-6405-10-30 PMC3874682

[22] Krishnan S, Dunbar MS, Minnis AM, Medlin CA, Gerdts CE, Padian NS. Poverty, gender inequalities, and women’s risk of human immunodeficiency virus/AIDS. Ann N Y Acad Sci 2008; 1136: 101–110. 10.1196/annals.1425.013 17954681 10.1196/annals.1425.013 PMC2587136

[23] Dugassa BF. Women’s rights and women’s health during HIV/AIDS epidemics: the experience of women in sub-Saharan Africa. Health Care Women Int, 2009; 30: 690–706. 10.1080/07399330903018377 19575321 10.1080/07399330903018377

